# 40-Hz auditory steady-state responses and the complex information processing: An exploratory study in healthy young males

**DOI:** 10.1371/journal.pone.0223127

**Published:** 2019-10-07

**Authors:** Vykinta Parciauskaite, Aleksandras Voicikas, Vytautas Jurkuvenas, Povilas Tarailis, Mindaugas Kraulaidis, Evaldas Pipinis, Inga Griskova-Bulanova

**Affiliations:** 1 Vilnius University, Institute of Biosciences, Vilnius, Lithuania; 2 Vilnius University, Institute of Psychology, Vilnius, Lithuania; Universidad de Salamanca, SPAIN

## Abstract

Electroencephalographic (EEG) activity in the gamma (30–80 Hz) range is related to a variety of sensory and cognitive processes which are frequently impaired in schizophrenia. Auditory steady-state response at 40-Hz (40-Hz ASSR) is utilized as an index of gamma activity and is proposed as a biomarker of schizophrenia. Nevertheless, the link between ASSRs and cognitive functions is not clear. This study explores a possible relationship between the performance on cognitive tasks and the 40-Hz ASSRs in a controlled uniform sample of young healthy males, as age and sex may have complex influence on ASSRs. Twenty-eight young healthy male volunteers participated (mean age ± SD 25.8±3.3) in the study. The 40-Hz click trains (500 ms) were presented 150 times with an inter-stimulus interval set at 700–1000 ms. The phase-locking index (PLI) and event-related power perturbation (ERSP) of the ASSR were calculated in the 200–500 ms latency range, which corresponds to the steady part of the response. The Psychology Experiment Building Language (PEBL) task battery was used to assess five cognitive subdomains: the Choice response time task, the Stroop test, the Tower of London test, the Lexical decision task and the Semantic categorisation task. Pearson‘s correlation coefficients were calculated to access the relationships; no multiple-test correction was applied as the tests were explorative in nature. A significant positive correlation was observed for the late-latency gamma and the mean number of steps in the Tower of London task reflecting planning and problem-solving abilities. These findings support the concept that 40-Hz ASSR might highlight top-down mechanisms which are related to cognitive functioning. Therefore, 40-Hz ASSRs can be used to explore the relationship between cognitive functioning and neurophysiological indices of brain activity.

## Introduction

Accumulating evidence suggests that electroencephalographic (EEG) activity in the gamma (30–80 Hz) range is related to the information processing associated with a variety of sensory and cognitive processes such as perception [1,2], attention [3–7], memory [8–11], response speed [12], object recognition [13], and language processing [14–16]. Many of the above mentioned aspects of cognitive functioning are impaired in schizophrenia (SZ) [17], with information processing speed decline being especially important in this condition [18]. Along with poor performance on cognitive tasks, the impaired gamma oscillations were frequently reported in patients [19], suggesting that cognitive alterations, as observed in SZ, can be attributed to the deficiency within networks supporting gamma activity [20]. The presence of gamma-range activity across spatial scales and cognitive operations suggests that the study of these oscillatory responses in the scalp-recorded EEG may shed the light on the functional integrity of neural circuits [21], where synchronization seems to play a critical role in the information processing [20].

The auditory steady-state response (ASSR) is the electrical response of the brain to regularly repeating auditory stimuli [22] that is used to test the ability to generate gamma frequency range (30–80 Hz) activity in patients with neuropsychiatric disorders [23,24]. The 40-Hz ASSR is proposed to serve as a potential biomarker of schizophrenia [21,25], as the impairment of the gamma-range ASSRs is frequently reported in patients [26–28], subjects at ultra-high risk [29] and relatives [27]. However, there is no firm conclusion on the functional significance of ASSRs. Some authors suggest it is a sensory response, reflecting the integrity of auditory circuits [23,26,30,31], others see ASSRs as a reflection of rather global synchronization of neural activity over with the external environment [29,32,33].

In line with the latter view, some evidence supports the link between gamma-range ASSRs and cognitive functions. First, the wide thalamo-cortical networks are involved in the generation of ASSRs [34–36], though the main contribution is from auditory cortex [37]. Second, 40-Hz ASSRs are modulated by arousal [38–40] and attention [41–43], i.e., states that are tightly associated with cognitive functioning [44]. Third, the gamma-range ASSRs were shown to correlate with the degree of cognitive decline in Alzheimer’s disease and Mild Cognitive Impairment patients [45]. In SZ patients ASSRs correlated with working memory [27,32,46], attention [29], reasoning and problem-solving abilities [47], metacognition and insight [48]. The above-mentioned findings suggest that gamma-range ASSRs (and 40-Hz ASSR in particular) may reflect the neurobiological mechanisms underlying some cognitive processes.

However, the analysis of the relationship between parameters of 40-Hz ASSRs and the basic cognitive abilities is limited to a few studies. Of those, several studies failed to show any correlation between ASSR and cognitive parameters in both patients and controls [49,50] or the relationships obtained in patients were not observed in the healthy control sample, i.e., association between ASSRs and working memory [32,49] and attention [29]. These results would suggest that gamma-range ASSRs do not necessarily require the engagement of neural networks associated with higher cognitive functions [32,49]. However, the studied samples were heterogeneous, both in respect to participants age and gender composition. The modulating effect of these factors on ASSRs was shown previously [51,52]. Thus, the relationship between cognitive functioning and networks underlying the generation of 40-Hz ASSR needs to be investigated further in controlled uniform groups to foster its usage as an individual biological marker for disturbed functioning in neuropsychiatric disorders [49,53]. Additionally, previous ASSR studies assessed the response over the central frontal locations only [27,29,32,48–50]. However, 40Hz ASSRs were reported to be somewhat larger over the right hemisphere [54,55]—either due to a generally higher right side activation with auditory stimulation [56] or due to the anatomic features of the left temporal regions causing left signal cancellation/distortions [57]. Thus, the assessment of associations between ASSRs over the left and right frontal regions and cognitive performance might provide additional information.

The present study aims to explore the possible relationship between the complex information processing and the 40-Hz ASSRs in a uniform sample of young healthy males. The complex information processing refers to the most basic operations of the human mind that are essential both for higher cognitive abilities and in everyday living [18]. The term relates to the mechanisms within the brain systems occurring during higher cognitive processes, rather than the content of the information per se [54] and different aspects (i.e., attention, problem-solving, lexical and semantic decisions) may be assessed by standardized neurobehavioural tests [53]. We hypothesized that parameters of 40-Hz ASSR—phase-locking value and event-related spectral perturbation—would positively correlate with information processing measures—information processing speed and efficiency. This assumption is based on the fact that synchronisation is crucial when subjects engage in faster and more complex cognitive processes [58,59].

## Methods

### Subjects

Thirty healthy non-smoking right-handed males (females were excluded because of the potential influence of hormonal fluctuations [60]) participated in the study. Subjects were asked to abstain from alcohol for 24 h prior to the testing. Also, they were asked not to consume nicotine and caffeine-containing drinks at least one hour prior to the experiment. Exclusion criteria for the study were any reported neurological disorder or known hearing problems. The hearing thresholds of all subjects were within the normal range (<25dB HL at octave frequencies). One subject was excluded from further analysis due to the bad quality of EEG recording and one—due to a technical issue with behavioural assessment. The final sample consisted of 28 participants (mean age ± SD 25.8±3.3]. The study was a part of larger project approved by the Vilnius Regional Biomedical Research Ethics Committee and all participants gave their written informed consent.

### Cognitive tasks

Each participant was given a brief description of the procedure prior to the testing. The Psychology Experiment Building Language [61] based task battery [62] consisting of the Choice response time task (CRT), the Stroop test (SOO), the Tower of London test (TOL), the Lexical decision task (LDT) and the Semantic categorisation task (SCT) was used to assess different aspects of information processing speed.

Reaction times (RTs), number of errors and response efficiency (as a mean reaction time divided by the proportion of correct responses on the test) were used as the outcome measures in the Choice response time task and Stroop test in all three conditions (congruent, incongruent and neutral). The CRT task was used to measure reaction time and psychomotor information processing speed [61,63]; SOO was used to evaluate executive function, attention, inhibition, and complex information processing speed [64]. The mean performance times, mean steps taken to perform the task and mean move time in the Tower of London test were used to measure executive function, planning speed, problem-solving and complex information processing speed [65]. The RTs, number of correct responses and efficiency scores were used to evaluate lexical memory and processing speed [62] in the Lexical decision task and semantic processing and information processing speed [66] in the Semantic categorisation task (SCT). Each task was precluded by the practice trial.

### Stimulation

The 40-Hz click stimulation trials lasted 500 ms and consisted of 20 identical clicks. Each 40-Hz trial was presented 150 times with an inter-stimulus interval set at 700–1000 ms. The auditory stimuli were generated by Arduino Uno microcontroller (^®^Arduino,https://www.arduino.cc/) and presented binaurally through Sennheiser HD 280 PRO earphones; sound pressure level was adjusted to 60 dB with a DVM 401 dB meter (Velleman, USA). Participants were asked to focus on stimulation and try to fixate their gaze at the fixation cross on the computer screen in front of them.

### EEG recording

EEG was recorded with an ANT device (ANT Neuro, The Netherlands) and a 64 channel WaveGuard EEG cap (International 10–20 System) with Ag/AgCl electrodes. Mastoids were used as a reference; the ground electrode was attached close to Fz. Impedance was kept below 20 kΩ and the sampling rate was set at 2048 Hz. Vertical and horizontal electro-occulograms (VEOG and HEOG) were recorded from above and below the left eye and from the right and left outer canthi.

### EEG processing

The off-line processing of EEG data was performed in EEGLAB and ERPWAVELAB for MatLab [62,63]. The power-line noise was removed using multi-tapering and Thomas F-statistics as implemented in CleanLine plugin for EEGLAB. Data were visually inspected and channels with substantial noise throughout the recording were manually rejected. An independent component analysis (ICA) was performed on the remaining channels with the ICA-implementation of EEGLAB (‘runica’ with default settings) and independent components related to eye movements were removed.

Epochs of 1200 ms were created starting at 500 ms prior to the stimulus onset and lasting for 700 ms post-stimulus onset; the epochs were further inspected for remaining artefacts. The data were baseline-corrected to the mean of the pre-stimulus period. A wavelet transformation (complex Morlet wavelet; frequencies represented from 1 to 120 Hz, with 1 Hz intervals between each frequency) was performed.

Phase-locking index (PLI), corresponding to the phase consistency over epochs, was calculated [67]. The event-related spectral perturbation (ERSP), indicating event-related changes in power relative to a pre-stimulus baseline, was also used as this measure is commonly applied in clinical ASSR-related studies [67]. The calculations were made according to the formulas

$$ITPC\left(c,f,t\right)=\frac{1}{N}\overset{N}{\underset{n}{\sum }}\frac{X\left(c,f,t,n\right)}{\left|X\left(c,f,t,n\right)\right|}$$

$$ERSP(c,f,t)=\frac{1}{N}\sum_{n}^{N}{\left|X(c,f,t,n\right|}^{2}$$

where for every channel c, frequency f and time point t a measure is calculated by taking time-frequency decomposition X of each trial n.

The mean PLIs and ERSPs were extracted focusing on the frequency window of 35–45 Hz by averaging the data over 200–500 ms. Data were baseline-corrected by subtracting the mean of the pre-stimulus period of 200 ms prior to the stimulus onset. In the next step PLI and ERSP values were grouped for the left (F3, F1, FC1, C1, FC3, C3), central (Fz, FCz, Cz), and right (F4, F2, FC2, C2, FC4, C4) regions.

### Statistics

Pearson‘s correlation coefficients were calculated to assess the relationship between mean PLI and ERSP values of the 40-Hz ASSR and measures of cognitive tasks. P values less than 0.05 were regarded as significant and no multiple-test correction was applied as the tests were exploratory in nature. The PLI and ERSP values between the regions (left vs. center vs. right) were compared by means of one-way ANOVA with subsequent post hoc tests. Statistical evaluation was performed using SPSSv20 (SPSS Inc., Chicago, Illinois, USA).

## Results

### Auditory steady-state responses

**Fig 1:**
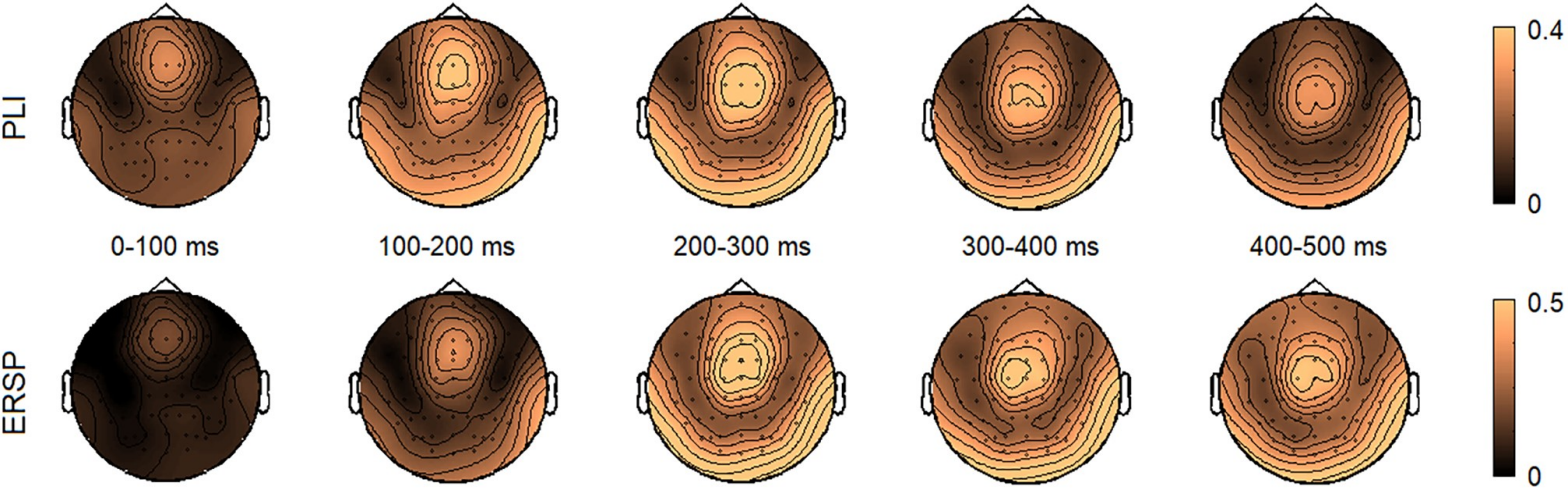
The topographies of PLI (upper panel) and ERSP (lower panel) within 0–500 ms time window in 100 ms bins in response to 40-Hz stimulation.

**Fig 2:**
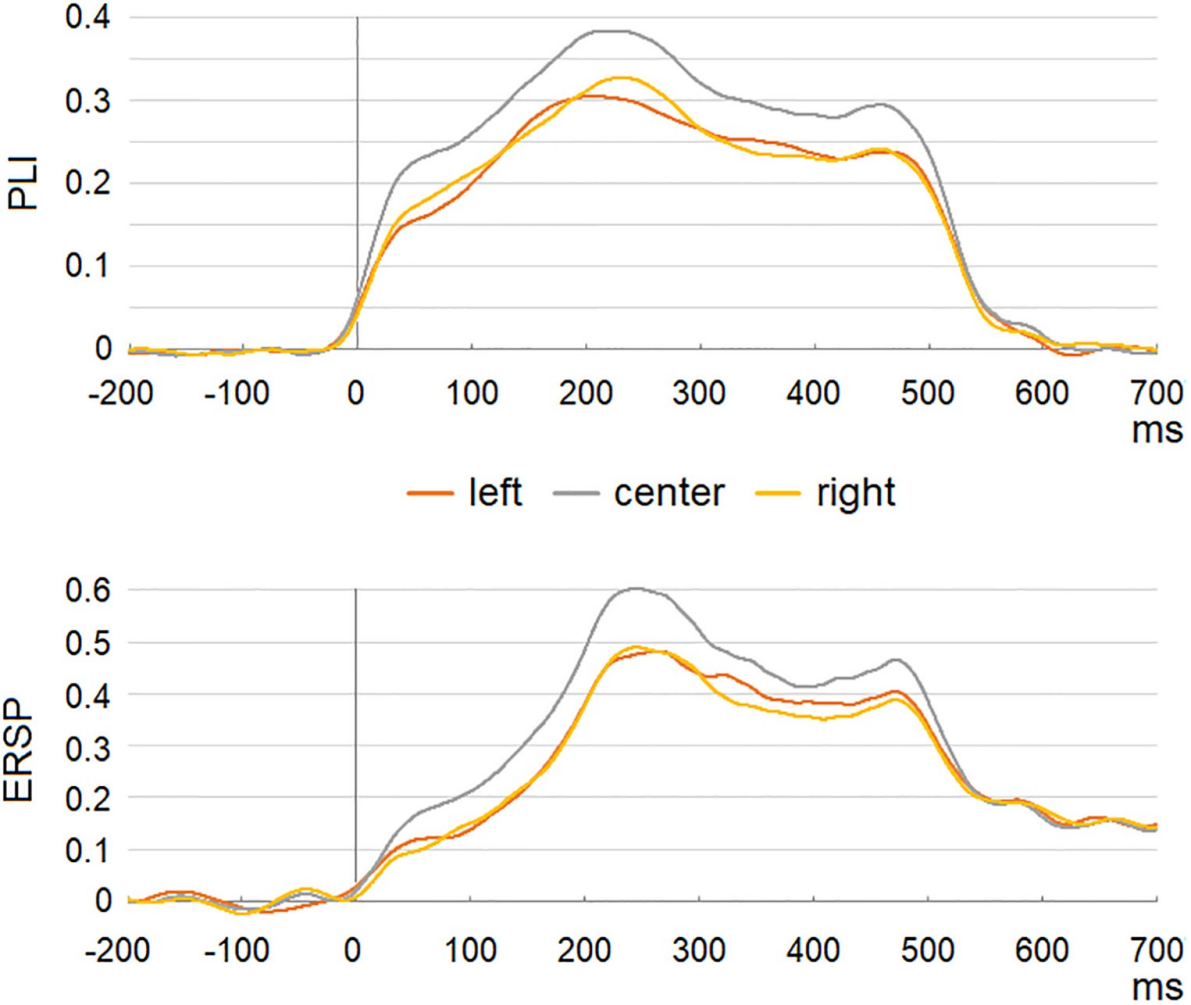
Time course of PLIs (upper panel) and ERSPs (lower panel) for the left, central and right regions in response to 40-Hz stimulation.

The topographies of PLIs and ERSPs in response to 40-Hz stimulation within 0–500 ms range in 100 ms windows are presented in Fig 1. The steady-state part of the response was established at around 200 ms and lasted till the end of stimulation. Analyses were performed on this fronto-central activation in 200–500 ms window corresponding to previous reports [32,68,69]. The time courses of PLI and ERSP values separately for left, central and right regions are plotted in Fig 2. The means and standard deviations of PLIs and ERSPs for the left, right and central regions are presented in Table 1. Somewhat larger PLI and ERSP values were observed over the central region, however differences were insignificant.

**Table 1:** Means and standard deviations of PLIs and ERSPs values during the 40-Hz stimulation for the left, central and right regions.

Site	Left		Center		Right	
	mean	SD	mean	SD	mean	SD
**PLI**	0.26	0.13	0.32	0.12	0.26	0.11
**ERSP**	0.42	0.24	0.50	0.23	0.41	0.18

### Cognitive tasks

Performance on cognitive tasks was similar to the results of previous research [66]. Means and SDs of quantified indices are presented in Table 2.

**Table 2:** Means and standard deviations of cognitive indices.

		mean	SD
**Choice response time task**	mean RT	387.12	49.92
	error	0.57	0.84
	efficiency	392.6	48.55
**Stroop test**	congruent mean RT	817.49	162.29
	congruent error	0.54	0.79
	congruent efficiency	836.88	166.22
	incongruent mean RT	985.26	213.47
	incongruent error	1.68	1.63
	incongruent efficiency	1072.52	281.78
	neutral mean RT	878.65	196.68
	neutral error	0.68	0.90
	neutral efficiency	902.8	189.82
**Tower of London task**	mean time	15452.73	5238.5
	mean move time	2057.72	677.16
	mean moves	60.68	6.0
**Lexical decision task**	mean time	1395.61	306.72
	error	2.79	1.83
	efficiency	1562.97	392.97
**Semantic categorisation task**	mean time	744.08	89.0
	error	0.32	0.55
	efficiency	750.14	89.29

### Correlations

**Fig 3:**
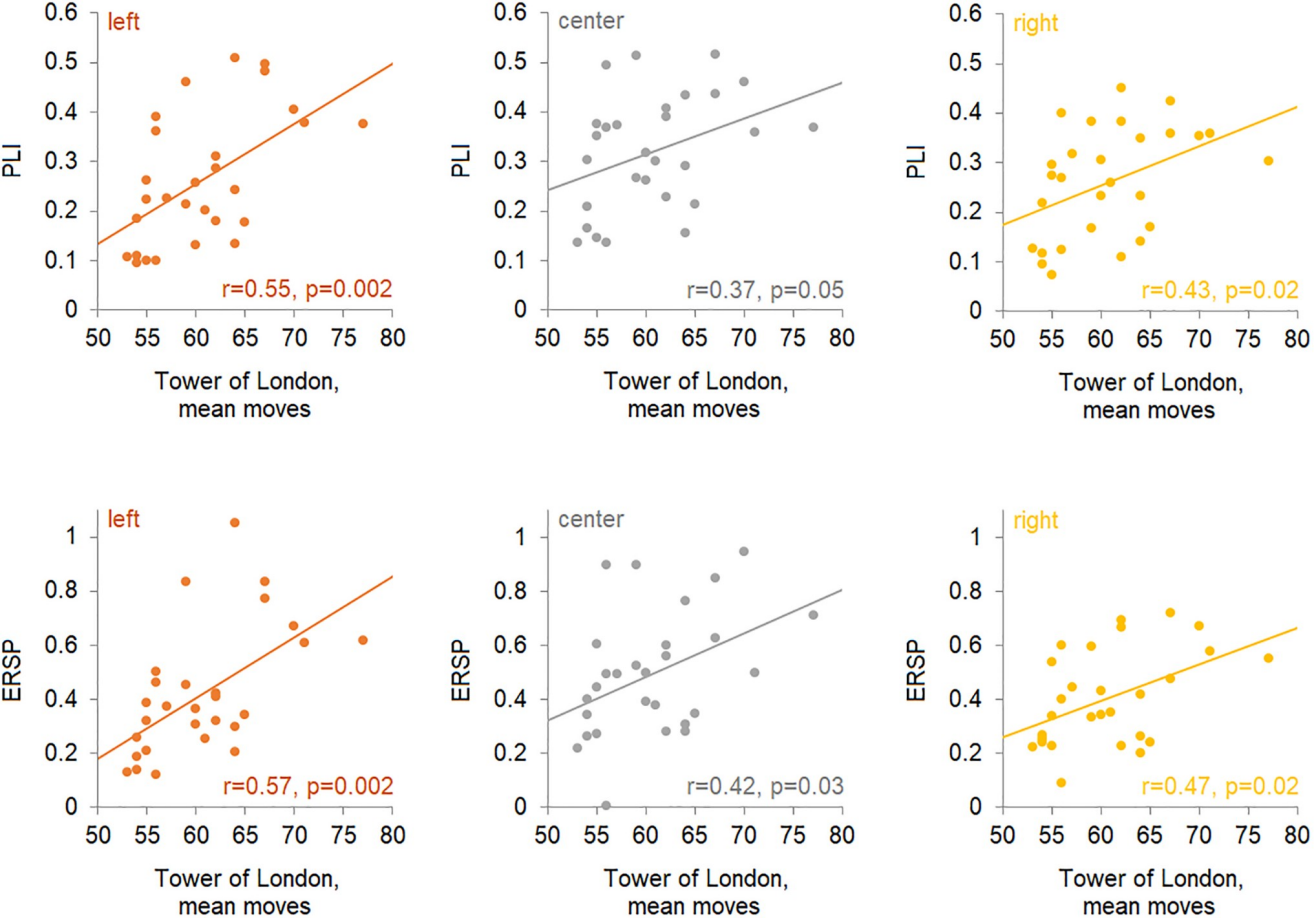
Scatterplots of PLIs and ERSPs against mean moves in the Tower of London Task. Plots are presented separately for the left, central and right regions.

Pearson’s correlation coefficients and corresponding p values for correlation between PLIs/ERSPs in response to the 40-Hz stimulation and cognitive indices are presented in Table 3. The significant correlations between the mean number of steps on the Tower of London task and PLI/ERSP values were observed for the left (PLI: r = 0.55, p < 0.01; ERSP: r = 0.57, p < 0.01), center (PLI: r = 0.37, p = 0.05; ERSP: r = 0.42, p = 0.03) and right regions (PLI: r = 0.43, p = 0.02; ERSP: r = 0.46, p = 0.01). Scatterplots of ERSPs against mean moves in the Tower of London Task are presented in Fig 3.

**Table 3:** Correlation coefficients and corresponding p values between 40-Hz ASSR parameters and cognitive task scores.

		PLI left		PLI center		PLI right		ERSP left		ERSP center		ERSP right	
		r	p	r	p	r	p	r	p	r	p	r	p
**Choice response time task**	mean RT	0.28	0.15	0.18	0.37	0.32	0.10	0.13	0.50	0.17	0.38	0.30	0.12
	error	-0.14	0.48	-0.06	0.75	-0.04	0.83	-0.09	0.64	-0.13	0.51	-0.18	0.35
	efficiency	0.27	0.17	0.17	0.38	0.32	0.09	0.12	0.53	0.16	0.42	0.28	0.14
**Stroop test**	congruent mean RT	0.24	0.22	0.07	0.72	-0.06	0.76	0.32	0.10	0.18	0.35	0.01	0.96
	congruent error	-0.14	0.48	-0.31	0.11	-0.30	0.12	-0.10	0.60	-0.34	0.08	-0.35	0.07
	congruent efficiency	0.21	0.28	0.02	0.94	-0.11	0.58	0.30	0.13	0.12	0.54	-0.05	0.79
	incongruent mean RT	0.23	0.23	0.17	0.39	0.09	0.65	0.28	0.15	0.26	0.17	0.16	0.41
	incongruent error	0.25	0.20	0.13	0.51	0.04	0.83	0.37	0.06	0.22	0.26	0.05	0.82
	incongruent efficiency	0.27	0.17	0.18	0.37	0.08	0.67	0.34	0.07	0.29	0.13	0.14	0.47
	neutral mean RT	0.28	0.15	0.18	0.35	0.14	0.48	0.34	0.08	0.33	0.08	0.16	0.41
	neutral error	-0.31	0.11	-0.31	0.11	-0.25	0.21	-0.32	0.10	-0.40	0.03	-0.34	0.08
	neutral efficiency	0.24	0.22	0.14	0.49	0.11	0.59	0.29	0.13	0.28	0.15	0.11	0.58
**Tower of London task**	mean time	0.08	0.70	-0.03	0.87	-0.02	0.90	0.11	0.56	-0.01	0.97	-0.15	0.46
	mean move time	0.31	0.11	0.30	0.11	0.20	0.30	0.32	0.10	0.29	0.13	0.14	0.48
	mean moves	0.55**	<0.01	0.37	0.05	0.43*	0.02	0.57**	<0.01	0.42*	0.03	0.46*	0.01
**Lexical decision task**	mean time	0.17	0.38	0.08	0.67	0.19	0.34	0.17	0.39	0.07	0.74	0.15	0.44
	error	-0.01	0.97	-0.03	0.86	0.10	0.62	-0.07	0.72	-0.02	0.90	0.03	0.89
	efficiency	0.14	0.47	0.08	0.70	0.22	0.25	0.11	0.58	0.05	0.78	0.17	0.39
**Semantic categorisation task**	mean time	0.24	0.22	0.17	0.37	0.27	0.16	0.22	0.25	0.13	0.52	0.15	0.44
	error	-0.06	0.76	-0.22	0.27	-0.24	0.21	0.05	0.79	-0.15	0.46	-0.21	0.29
	efficiency	0.24	0.23	0.15	0.44	0.24	0.21	0.23	0.23	0.11	0.56	0.13	0.51

* Correlation is significant at the 0.05 level,

** correlation is significant at the 0.01 level.

## Discussion

The involvement of the gamma activity in various cognitive functions is a well-documented finding [9,70] as are the alterations of gamma-band in schizophrenia (SZ) [20,21,23]. However, the extent of the relationship between various cognitive domains and gamma responses elicited with 40-Hz stimulation remains unclear. We explored the possible relationship between cognitive functioning indices in the dimensions that are more frequently reported to be altered in SZ and the measures of 40-Hz ASSR in a healthy uniform group of male subjects. The phase-locking properties and event-related changes in power relative to a pre-stimulus baseline were evaluated. The only significant correlation was observed for the steady part of 40-Hz ASSR and the mean number of moves on the Tower of London task (Fig 3). The association was evident both for PLI and for ERSP over the left, center and right regions, and somewhat stronger correlation was observed for the left side response.

The Tower of London (ToL) task reflects executive function, planning and problem-solving [65,71]. Unexpectedly, the stronger and more synchronized responses were observed in subjects who performed more steps in ToL, i.e., were less efficient on the task. This contradicts recent observation by Sun et al. [47], who showed a positive correlation between another test for reasoning and problem solving—Mazes test from the Matrics Consensus cognitive battery (MCCB) [72]—and the phase-locking properties of 40-Hz ASSR in both SZ patients and controls, indicating better performance in subjects with more synchronized ASSRs. However, the discrepancy between the tests and in the scoring approaches in used ToL variations makes comparison to previous results difficult. The ToL task, as implemented in Brief Assessment of Cognition in Schizophrenia (BACS) battery [73], was previously used by Tada et al. [29]. Although they did not test healthy controls, the relationship between measures of 40-Hz ASSR and a number of correct responses as an outcome score in their ultra-high risk and first episode SZ subjects was not observed. Earlier, Diez et al. [74] reported a negative correlation between the total gamma power (35–45 Hz) while performing P300 oddball task and the Tower of London task scores on BACS battery in both SZ patients and their relatives. However, no association in healthy controls was detected. The ToL outcome, as implemented in BACS, is based on the number of correct responses (ranging 0–22), where higher scores indicate better performance. Thus, a negative correlation between gamma power and ToL scores in a study by Díez et al. [75] would also indicate poorer performance in subjects with higher gamma, similar to our observation. Díez et al. [74] obtained comparable results when evaluating gamma noise power—the loading of frontal lateralized component was significantly and inversely related to problem-solving in patients. Diez et al. [74] have speculated that higher gamma in patients and their relatives is a sign of overactivation that prevents good performance on ToL. This might be the case in our subjects, who were able to generate strong ASSRs, i.e., were more reactive to gamma stimulation, but were less efficient in ToL.

Cazalis et al. [76] have suggested that superior performers and standard performers in ToL could potentially elaborate different strategies, as indicated by different patterns of activation. Authors proposed that standard performers might use a larger working memory span compared to superior performers [76]. We did not evaluate working memory in our group, however, prior research has indicated a positive relationship between working memory and measures of ASSRs [29,32,46]. It is possible that a positive relationship between 40-Hz ASSRs and mean move times in ToL is indicating the working-memory related aspects of the task. This assumption should be tested in further studies.

We did not evaluate some domains that were shown to be correlated to 40-Hz ASSR measures before, like abstract verbal reasoning [27] or working memory [46]. These associations should be tested in future research. However, the discrepancies in the observed relationships between performance on cognitive tests and 40-Hz ASSRs are inconsistent even when the same stimulation, analysis and scoring settings are elaborated. For example, Rass et al. [27] observed a positive relationship between gamma range ASSRs and verbal reasoning in SZ patients, their relatives and control subjects; however, in a sample of bipolar patients and matched controls no such association was observed in a study with similar experimental settings [50]. Light et al. [32] observed a positive correlation with working memory in SZ patients; however, Kirihara et al. [49], using the same stimulation settings and assessment tools, failed to show any relationship in their group. These results suggest that relationships might depend on the individual characteristics of the groups tested.

Only young male subjects were enrolled in the current study, thus any potential gender-related aspects were avoided. Some of the employed cognitive tasks are known to be differently performed by males and females [77] and 40-Hz ASSRs were previously shown to differ between sexes [49,51]. This could have affected the observed relationships in prior reports. Another factor potentially modulating the relationship between cognitive domain and electrophysiological indices is subjects’ age. The decline in performance on cognitive tasks is a well-established observation [78] and changes of ASSRs with age were also showed [52,79]. The age representation of our sample was narrow, thus observed relationships were not affected by potential aging effects; this could also cause less variable data and thus hamper some associations potentially explaining why we did not observe any other correlations. Nevertheless, based on the prior reports and our current observation, the 40-Hz ASSRs were related to more complex tasks having executive aspect, and supporting the notion that synchronisation is crucial when subjects engage in faster and more complex cognitive processes [58,59]. It is possible that other tasks employed in this study were not complex enough. This feature should be addressed further.

Auditory stimulation is frequently used to test gamma activity as it produces the strongest EEG responses in the gamma range [79–81]. However, it is unclear whether auditory sensory modality is the most optimal for detecting gamma-band abnormalities [82]. Thus, further studies should include stimuli of other modalities to test associations of periodic responses to performance in cognitive domain.

Finally, ASSR assessment approach could influence the observed relationships. As detected in the current study, weak correlations between measures over the central locations (that are commonly used for the assessment of associations to cognitive symptoms (for example 27,29,49] and ToL were observed, opposite to the lateralized left and right sides. This is in line with Díez et al. [72,73], who reported relationship to responses from lateralized locations in their groups. Thus, lateral locations should also be included in the evaluation of ASSRs in the future studies.

### Conclusions

In this study, aiming to assess the relationship between performance on cognitive tasks and measures of 40-Hz ASSR, the positive correlation between the strength and synchronicity during the steady part (200–500 ms) of the response and the mean number of steps on the Tower of London task was observed. This association potentially indicates that the late-latency gamma in response to auditory 40-Hz stimulation might index abilities for planning and problem-solving. The finding supports the concept that 40-Hz ASSR might highlight the top-down mechanisms which are related to cognitive functioning and can be a useful tool to explore the relationship between cognitive functioning and neurophysiological indices of brain activity.
